# Axillary Treatment Management in Breast Cancer during COVID-19 Pandemic (Association between ACOSOG Z0011 Criteria and OSNA Test)

**DOI:** 10.3390/jpm13020241

**Published:** 2023-01-29

**Authors:** Giacomo Anedda, Federico Cappellacci, Gian Luigi Canu, Stefania Farris, Pietro Giorgio Calò, Massimo Dessena, Fabio Medas

**Affiliations:** 1 Department of Surgical Sciences, University of Cagliari, 09124 Cagliari, Italy; 2 Polispecialistyc Surgery Unit, Policlinico Universitario “Duilio Casula”, 09042 Cagliari, Italy

**Keywords:** breast surgery, breast cancer, axillary lymph node status, sentinel lymph node biopsy, de-escalating surgery, OSNA method, ACOSOG Z011

## Abstract

The outbreak of the SARS-COVID-2 pandemic (COVID-19) had a significant effect on the organisation of healthcare systems. Surgical units saw a significant reduction in the volume of surgical procedures performed, with lengthening waiting lists as a consequence. We assessed the surgical activity in relation to breast cancer that took place at the University Hospital of Cagliari, Italy, from February 2018 to March 2022. Two phases were identified based on the epidemiological circumstances: Phase 1—February 2018 to February 2020; Phase 2—March 2020 to March 2022. The surgery performed in the two phases was then compared. All the patients in our sample underwent a breast surgical procedure involving a lymph node biopsy using OSNA associated with the ACOSOG Z0011 criteria. In the study period overall at our facility, there were 4214 procedures, 417 of which involved breast surgery. In Phase 2, 91 procedures were performed using the OSNA method and ACOSOG Z0011 criteria, enabling the intraoperative staging of axillary nodes. Axillary treatment in breast cancer using this approach resulted in a significant reduction in the number of reoperations for the radicalisation of metastatic sentinel lymph nodes.

## 1. Introduction

The COVID-19 pandemic emerged in 2020. In response, the Italian government, like many others, sought to limit the rising number of infections and deaths by imposing a strict lockdown regime. This began on 8 March 2020, and comprised a series of restrictive measures that ordered the population to stay at home save for defined, very limited purposes. Three days later, the World Health Organization (WHO) officially declared that COVID-19 was a global pandemic. The restrictions imposed encompassed a large part of the healthcare system, which had to concentrate and apply resources, both material and human, to the fight against the disease. These curtailed (to the minimum necessary) the access of healthcare personnel to healthcare facilities and saw a drastic reduction in scheduled care over the provision of emergency assistance. Surgical procedures were particularly affected, which not only lengthened waiting lists, but also meant delayed diagnoses and/or follow-ups. On 31 March 2022, the Italian government declared the COVID-19 state of emergency to be at an end [1].

In breast cancer patients with clinically node-negative (cN0) disease, a sentinel lymph node biopsy (SLNB) is currently the gold standard for assessing their axillary status. For those with early breast cancer and lymph node metastases, axillary treatment is widely recommended to reduce the risk of axilla recurrence and improve survival [2,3]. Examination of the axillary lymph nodes must be performed histologically once the surgery has been completed. Further axillary dissection, if required, then necessitates a second surgical procedure. The period between a quadrantectomy or mastectomy during which the SLNB is conducted and axillary dissection is typically about a month. The sentinel lymph node (SLN) can also be evaluated using a real-time molecular technique known as the reverse transcription-polymerase chain reaction (RT-PCR), where a sample is examined in a specialist laboratory with an execution time of about three hours. When there is metastatic involvement of the sentinel lymph node, it is also possible to perform an intraoperative histological evaluation, which allows the surgeon to proceed with the axilla dissection at the same time as the quadrantectomy or mastectomy. The length of this procedure varies and depends on numerous factors, but it is generally about an hour. The SLNB can be performed using the one single nucleotide amplification (OSNA) method, which enables a quick evaluation of the involvement of the sentinel lymph nodes and an intraoperative diagnosis [4,5].

A Phase 3 randomised clinical trial conducted by the American College of Surgeons Oncology Group Z0011 (ACOSOG- Z0011) involving women with T1 or T2 invasive primary breast cancer, no palpable axillary adenopathy and one or two sentinel lymph nodes containing metastases, found that overall survival in patients treated with sentinel lymph node dissection alone was not inferior to that in those treated with axillary lymph node dissection [6,7,8].

The aims of our study were to evaluate: (1) whether the COVID-19 pandemic affected surgical procedures at our facility; (2) the differences between prepandemic and pandemic cases, particularly in relation to breast cancer; (3) if SLNB employing the OSNA method and the ACOSOG Z0011 protocol had value for axillary staging and treatment in cases of metastatic lymph nodes during a period when there were major restrictions on surgical procedures and patients’ access to hospitals.

## 2. Materials and Methods

In this study, we analysed the activity of the poli-specialist surgery unit of AOU Cagliari of the University Hospital of Cagliari, Italy, in the period from February 2018 to March 2022. Two phases were identified based on the epidemiological evolution of the COVID-19 pandemic and the measures imposed on the population by the Italian Government: Phase 1—February 2018 to February 2020; Phase 2—March 2020 to March 2022. We focused our attention on breast cancer surgery, comparing the patients operated on during the two periods, all of whom underwent a breast surgical procedure using the OSNA method and the ACOSOG Z0011 criteria.

In particular, we observed the breast surgery patients placed into the following groups based on the type of procedure they underwent: quadrantectomy, simple unilateral and bilateral mastectomy, and local lesion excision. For statistical purposes, a bilateral mastectomy was treated as a unilateral double mastectomy. All the patients who underwent a procedure using OSNA and the ACOSOG Z0011 protocol had: T1 (up to 2 cm) or T2 (>2 cm <5 cm) cancer based on tumour, node, and metastasis (TNM) staging; clinical or no palpable axillary adenopathy; ultrasound negative lymph nodes (pT1N0–pT2N0) [7,8]. A sample of axillary lymph adipose tissue was removed from all the patients (lymph node sampling) with almost three SLNs. To evaluate the status of the non-SLNs (NSLNs), the tissue was sent for investigation intraoperatively, even in cases where there were no metastases on the OSNA examination. This enabled us to verify the predictive value of SLNs analysed with OSNA for the status of the non-sentinel axillary lymph nodes in our study population. In accordance with the ACOSOG Z0011 protocol, all the patients who tested positive for more than two macrometastatic lymph nodes during the OSNA examination underwent a level I or II axillary lymphadenectomy. Their lymph nodes were sent for definitive histological assessment. Those who tested positive for two or fewer macrometastatic lymph nodes did not undergo any further procedures [4,5,7,8]. All the patients who underwent the examination of a single SLN with OSNA but from whom no additional axillary lymph adipose tissue was taken, and there was thus no further evaluation, were excluded from the study.

All patients underwent multidisciplinary evaluation both before and after the surgical procedure.

All the patients who underwent neoadjuvant chemotherapy were excluded. Currently, the OSNA method after neoadjuvant therapy has some validation studies [9,10,11,12]. The national guidelines on which our clinical pathway is based and this study do not provide the use of the OSNA method for all those patients who underwent neoadjuvant chemotherapy. The parameter pN taken as the measure of the largest tumour cell aggregate, not as the sum of single microclusters or isolated metastatic cells, should be considered to define macrometastases versus micrometastases. This parameter, defined above, gives rise to problems in the use of the OSNA molecular method, which could lead to an overstaging of lymph node involvement. The OSNA is a method based on the extraction of mRNA from a homogeneous lymph node, preventing the measurement of the major metastatic deposit [13,14]. Discordant data have been reported for the application of the OSNA method after therapy neoadjuvant; it should be noted that the use of the OSNA method does not allow for the evaluation of the post-treatment histological response, and our system is not calibrated to detect isolated tumour cell (ITC) [13,14]. The main problem using the ONSA method after neoadjuvant treatment is that the evaluation of histological lymph nodal asset after secondary treatment is lost. Moreover, the lymph node remnant after treatment is the only neoplastic cells residual from the entire surgery, resulting lost the opportunity for the immunohistochemical study [9,10,11,12]. Therefore, we had to exclude all those patients. For our national lineguide update we’are looking forward incoming focused studies, that we are involved with, that are dedicated directly to this particular class of patients.

Also excluded were patients for whom the data requested were not exhaustive and/or were incomplete.

The data reported in our study starts in 2018. In late 2019, the fifth edition of breast cancer classification was published by the World Health Organization (WHO), and it changed the anatomopathological nomenclature of cancer types [15]. Our pathological anatomy department has started to adopt the current nomenclature in the histological reports since 2020, leaving the old histological description as an additional note in the reports. Consequently, the nomenclature in the reports of our sample results is heterogeneous. In agreement with our pathological anatomy institute, we have changed the previous nomenclature to the current one reported in the WHO 5th edition. Moreover, we have considered it appropriate to maintain this double nomenclature as additional data in our database.

The assessment of the lymph nodes was performed using the Sysmex RD-100i, which allows the simultaneous analysis of up to four lymph nodes in about 30 min. The results were expressed in terms of the number of mRNA copies measured by the CK-19 marker. All the lymph nodes weighed more than 50 mg and were no less than 10 mm in size, as they could not be analysed if they did not comply with these parameters. The RT-PCR results obtained with the OSNA assay enabled us to distinguish macrometastases (pN1a: a diameter >2 mm, according to TNM staging) of >5000 copies and micrometastases (pN1mi: a diameter between 0.2 and 2 mm, according to TNM) of 250–5000 copies. Isolated tumour cells (ITCs) (pN0 i+ diameter <0.2 mm according to TNM, 2019) were not recognised [16,17].

For all the statistical analyses, continuous data were preliminarily evaluated with the D’Agostino–Pearson test to evaluate whether data were normally distributed. The results were reported as the mean ± standard deviation, and the Student’s t-test was used to test the differences between the two groups. The chi-squared test was used to compare categorical variables. Hypothesis tests were 2-sided, and the results were considered significant at *p* < 0.05. Statistical analyses were performed with MedCalc (MedCalc Software Ltd., Ostend, Belgium) vers. 20.123.

The study was approved by the Comitato Etico Indipendente (NP/2022/1373) from Azienda Ospedaliera Universitaria di Cagliari.

## 3. Results

We initially examined 4214 general surgery procedures conducted between February 2018 and March 2022, 417 of which were for breast cancer. Of these 417 cases: 200 (48.0%) involved a conservative quadrantectomy, 150 (35.9%) involved a simple mastectomy, 92 (22.1%) involved a unilateral, 29 (6.9%) involved a bilateral, and 96 involved a local lesion excision (23.0%). A total of 204 breast surgeries were performed in Phase 1 and 213 in Phase 2, representing a minimal increase (+4.4%) (Appendix A). There were no differences in the types of procedures performed in the two phases, save that the number of quadrantectomies decreased (52.2% vs. 43.9%) and the number of mastectomies increased (32.5% vs. 39.8%), even if this difference was not statistically significant (Table 1).

Of these 417 procedures, 166 patients were treated with the OSNA method and the ACOSOG Z0011 protocol. In particular, they had primary breast cancer (T1–T2) with clinically and ultrasound-negative lymph nodes underwent SLNB via the OSNA molecular method and had up to two affected NSLNs. Of the 166 patients, 163 were female, and three were male; one of the males had exotoxic cirrhosis and gynecomastia that developed into unilateral breast cancer, while the other two had primary male breast cancers. The average age was 62 years.

Infiltrating carcinoma, no special type (NST) was found in 98 patients; infiltrating lobular carcinoma was found in 29; ductal carcinoma in situ in 8; other types of carcinoma were found in 29 cases including mucinous carcinoma in 17, ductal-lobular infiltration in 7, and a clear cell tumour in 5. The tumour sizes ranged from 7 to 48 mm. There were no significant differences between the two phases in terms of the histotype or tumour size: infiltrating NST carcinoma—44 vs. 54; infiltrating lobular carcinoma—13 vs. 16; DCIS ductal carcinoma in situ—4 vs. 6. Other types: mucinous carcinoma—7 vs. 10; infiltrating ductal-lobular carcinoma—3 vs. 4; clear cell tumour—3 vs. 2 (Table 2).

There were 75 procedures carried out using OSNA and the ACOSOG Z0011 protocol in Phase 1 and 91 in Phase 2. During Phase 2, 91 procedures were performed using the OSNA method, enabling complete axillary staging to be conducted in a single operation. The Phase 1 group contained 17 cases that tested positive for macrometastases in more than two LNs (22.7%); in the Phase 2 group, there were 40 such cases (44.0%), and we proceeded to perform an axillary lymphadenectomy (Appendix B). The differences in the macrometastases’ positivity between the two phases were statistically significant for Phase 2, with 40 cases (44.0%) of positivity in more than two LNs versus 17 (22.7%) in Phase 1 (Table 3).

## 4. Discussion

**Figure 1:**
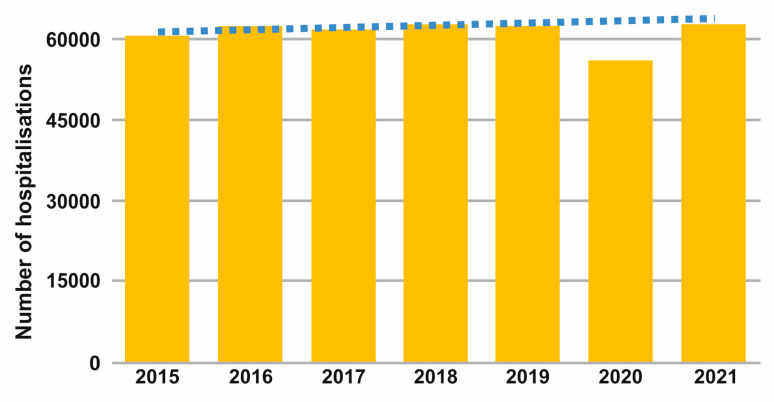
Number of hospitalisations for breast cancer in the period 2015–2020.

Breast cancer is the most commonly diagnosed and hospitalised malignancy and is the leading cause of cancer death in women of all age groups. In Italy, in the period before the COVID-19 pandemic, there had been a slight increase in the volume of surgical admissions for malignant tumours (+2.8%). In 2020, however, there was a significant reduction in the number of hospitalisations, amounting to approximately 6300 fewer cases than in 2019 (circa—11%) [17] (Figure 1).

The study sample is part of a regional population of over 1.5 million people. According to the national guidelines, this population would require at least one breast unit for every 250,000 inhabitants for the correct treatment of breast cancer [18]. Currently, in our region, only two breast units are accredited. It should be emphasised that the population examined lives on an island and, therefore, makes it difficult to move towards reference centres located in the national territory. This difficulty faced by the local population in accessing breast centres that can offer the right treatment has been made even more problematic during the pandemic, bringing out the differences between the centres that use recent methods and those that still use less recent ones.

The OSNA method was introduced in our hospital, not exclusively for breast cancer, in 2017 and has become commonly used since 2018 [19]. We are currently part of the national network of OSNA user methods; we are the only surgical department registered and the only one constantly using the OSNA method in a regional area. It should be remembered that during the most critical phases of the pandemic, all travels between regions were forbidden. That means our institute was the only hospital that could offer this procedure to the entire population of the region.

The pandemic has led to a profound reassessment of available resources. This has increased the gap in the possibilities of diagnosis and therapy between hospitals that have already adopted the most recent procedures and those that still have to update.

In our study period of four years, in terms of the differences between the study’s two phases in relation to breast cancer, we did not identify any reduction in operative volumes, contrary to national data. However, our centre has been experiencing a constant annual growth in cases of about 20% over the previous three years. This means that the practically stable number of surgical procedures performed in the study’s pre- and pandemic periods effectively represents an arrest in this trend, which is comparable with the national picture [20]. This is an important concept to be evaluated, especially for breast cancer, because that involves a considerable number of women. The challenge during the pandemic period was to offer quality care to all patients by limiting exposure to pandemic risk. International society and our national health care organisation have issued scalable recommendations to provide a framework to aid clinicians in prioritising essential cancer care dependent on the extent of the health crisis. These indications identify breast cancer patients where time is of the essence for the treatment of the disease [21,22].

In Phase 1, 91 procedures were performed using the OSNA method and the ACOSOG Z0011 protocol. This meant that complete staging of the axillary status was achieved in a single operation; it also meant shorter operating times (about 50 min) compared to extemporaneous histological procedures that take about one hour for results. We also considered ductal carcinoma in situ (DCIS) for this study because it underwent the sentinel lymph node biopsy with the OSNA method. This surgery occupies the operating room for a procedure that can be assimilated, in terms of time duration, to a quadrantectomy with sentinel lymph node biopsy. Moreover, the OSNA method gave us a clear status of axillary diagnosis, even if, for this type of tumour, there is no indication, other than theoretical, for axillary dissection. Therefore, the OSNA procedure allowed the best use to be made of the operating spaces available, with several surgeries that can be performed in the same session [23,24,25].

There was a difference in the procedures carried out in Phase 2 (more mastectomies and fewer quadrantectomies). This was because patients did not have easy access to hospital facilities or faced delays [23,25,26]. Moreover, it was not possible to conduct screening, leading to disease progression [24,25].

We also identified a significant increase in the positivity of axillary lymph nodes (20 vs. 40). This figure is far higher than the data in the literature and can also be explained by the fact that patients received medical attention later than in the prepandemic period [26,27,28]. This trend is ongoing, probably still as a result of the screening and diagnostic delays caused by access problems during the pandemic [29,30].

Numerous studies report a significant reduction in access to screening for breast disease. The data routinely collected by NHS England Cancer Waiting Time were analysed to compare activity for breast cancer in the first 6 months of 2020 compared to the same time period in 2019. The number of reports for suspected breast cancer was 28% lower, and the number of patients who received their first treatment for a breast cancer diagnosis was 16% lower. These data suggest that the number of breast cancers diagnosed during the first half of 2020 is not as low as initially feared, and a substantial proportion of the shortfall can be explained by the suspension of routine screening in March 2020 [31]. A second evaluation from a large retrospective study from a high-volume centre demonstrated that between the prepandemic period and March 2020 to March 2021, differences in the clinical stage between periods were relevant, with cancers from the mid-period (September 2020 to March 2021) showing the most advanced stage. A shift to a later stage was plausibly a result of delayed intervals in the early COVID period [32].

A crucial concept regarding the evaluation of the patients examined in this study will be the follow-up of patients with a single positive lymph node for macrometastasis where axillary lymphadenectomy has not been performed. Follow-up for invasive cancers is defined according to international guidelines: European Society for Medical Oncology (ESMO) recommends a visit every three or four months in the first two years, every six months from years three to five and then annually, followed by an annual ipsilateral (after breast conservative therapy) and/or contralateral mammography with ultrasound evaluation [33]. The National Comprehensive Cancer Network (NCCN) recommends history and physical exams one to four times per year as clinically appropriate for 5 years, then annually, and mammography every 12 months [34]. In our centre, we follow the most recent national guidelines (Associazione Italiana Oncologia Medica–AIOM) [13], which are based on international literature that recommends history and physical exams every three or six months in the first three years, then every six months or annually. Mammography should be performed one year after the mammography that made diagnosis. In women undergoing conservative surgery, mammography is recommended six months after the end of radiotherapy and then annually.

This situation will not only affect patients who have breast disease but will involve the entire population because it is estimated that diagnostic delays, treatment interruptions, and the worsening of current diseases will lead to a significant increase in public medical spending [35].

The period examined in our study started in March 2018, and the results of the first patients examined will be available in the first half of 2023. This condition puts us in the position of not being able to evaluate, even partially, the result of these procedures.

The application of the ACOSOG Z0011 protocol meant that reoperation was avoided in 41.5% of the 40 cases where there was positivity for macrometastases in more than two LNs, with axillary lymphadenectomy instead performed in a single procedure. Although this lengthened the operating time compared to an SLNB, it meant there was no need for a second operation. As a result, the operating time freed up, enabling a further 40 breast surgery procedures to be conducted, 18.6% of breast interventions of the total volume of planned interventions of the breast unit.

This study has some limitations. First, this is a single-centre, retrospective study. The sample examined is limited by the operating volume: our reference centre for this study is increasing its operating volume but still cannot be considered as a breast unit [13]. The study was performed in a local region where the pandemic hit particularly hard; the generalization of our results to other populations should be made carefully. Finally, the real incidence of disease recurrence cannot be estimated in our study: the average follow-up time is still too short to understand the clinical significance. Further studies with larger samples and involving more centres in the same region should be considered to have a clear picture of the pandemic period, and a review of follow-up results can improve this study.

## 5. Conclusions

The COVID-19 pandemic led to a decrease in the number of surgical procedures conducted at our facility, minimally including those for breast cancer. In addition, the status of patients at the time of their intraoperative diagnosis had also worsened. The axillary treatment of breast cancer using the OSNA method and the ACOSOG Z0011 protocol resulted in a significant reduction in the volume of reoperations required for the radicalisation of metastatic LNs, allowing more interventions to be performed, thus reducing the length of the waiting list and the impact of the pandemic on surgical activity. We wanted to focus on the association of the two methods that are used in our normal clinical practice but not in all regions’ hospitals that are involved with breast cancer, making it possible to limit the important difficulties that have arisen with the beginning of the pandemic. This is because, in the pandemic, our centre has maintained a practically constant number of surgical procedures for breast cancer, while an important reduction in surgical procedures was reported in all other pathologies that required surgery, both nationally and in our own institute [36]. Even today, the use of the OSNA method, especially in our region, is very limited. The application of this procedure would allow for better and more appropriate care. This helped identical volumes to be maintained, despite the difficulties of both managing resources and the impact of the pandemic on patients, who often faced delays due to the prioritisation of treatment for COVID-19.

## Figures and Tables

**Table 1:** Surgery procedures.

Surgery	Phase 1		Phase 2		Total		*p*
	*n.*	%	*n.*	%	*n.*	%	
Procedures	203		214		417		
Quadrantectomy	106	52.2	94	43.9	200	48.0	0.11
Single Mastectomy	40	19.7	52	24.3	92	22.1	0.31
Double Mastectomy	13	6.4	16	7.5	29	6.9	0.81
Others ^1^	44	21.7	52	24.3	96	23.0	0.96

^1^ Local lesion excision, recurrences, ink on tumour.

**Table 2:** Demography.

	Phase 1 (*n* = 75)	Phase 2 (*n* = 91)	Total	*p*
**Sex**				0.86
Female	74	89	163	
Male	1	2	3	
Age (years)	62.4 ± 14.2	61.8 ± 14.6	62.1 ± 14.4	0.82
BMI (Kg/m^2^)	26.3 ± 3.1	25.6 ± 3.4	26 ± 3.3	0.34
**ASA**				0.62
I	23	28	51	
II	42	46	88	
III	10	17	27	
**Menopausal status**				0.79
Premenopausal	29	37	66	
Postmenopausal	46	54	100	
Tumour size (mm)	20.5 ± 7.3	22.1 ± 8	21.5 ± 7.7	0.24
**Histological type**				0.98
NST	44	54	98	
Lobular	13	16	29	
DCIS	4	6	10	
Others	13	16	29	
mucinous	7	10	17	
ductal-lobular	3	4	7	
clear-cell	3	2	5	

**Table 3:** OSNA results.

	Phase 1		Phase 2		*p*
	*n.*	%	*n.*	%	
OSNA procedures	75		91		
Negative	40	53.3	35	38.5	<0.001
Positives	35	46.7	56	61.5	<0.001
Metastasis ^1^ < 2 LNs	18	24.0	16	17.6	0.95
Macrometastasis > 2 LNs	17	22.7	40	44.0	0.006

^1^ Micrometastasis or Macrometastasis.
